# Supplementary figures and images for: Impact of variability in cell cycle periodicity on cell population dynamics
Source: PLoS Comput Biol. 2023 Jun 20;19(6):e1011080. doi: 10.1371/journal.pcbi.1011080 (PMC10313040; doi:10.1371/journal.pcbi.1011080)

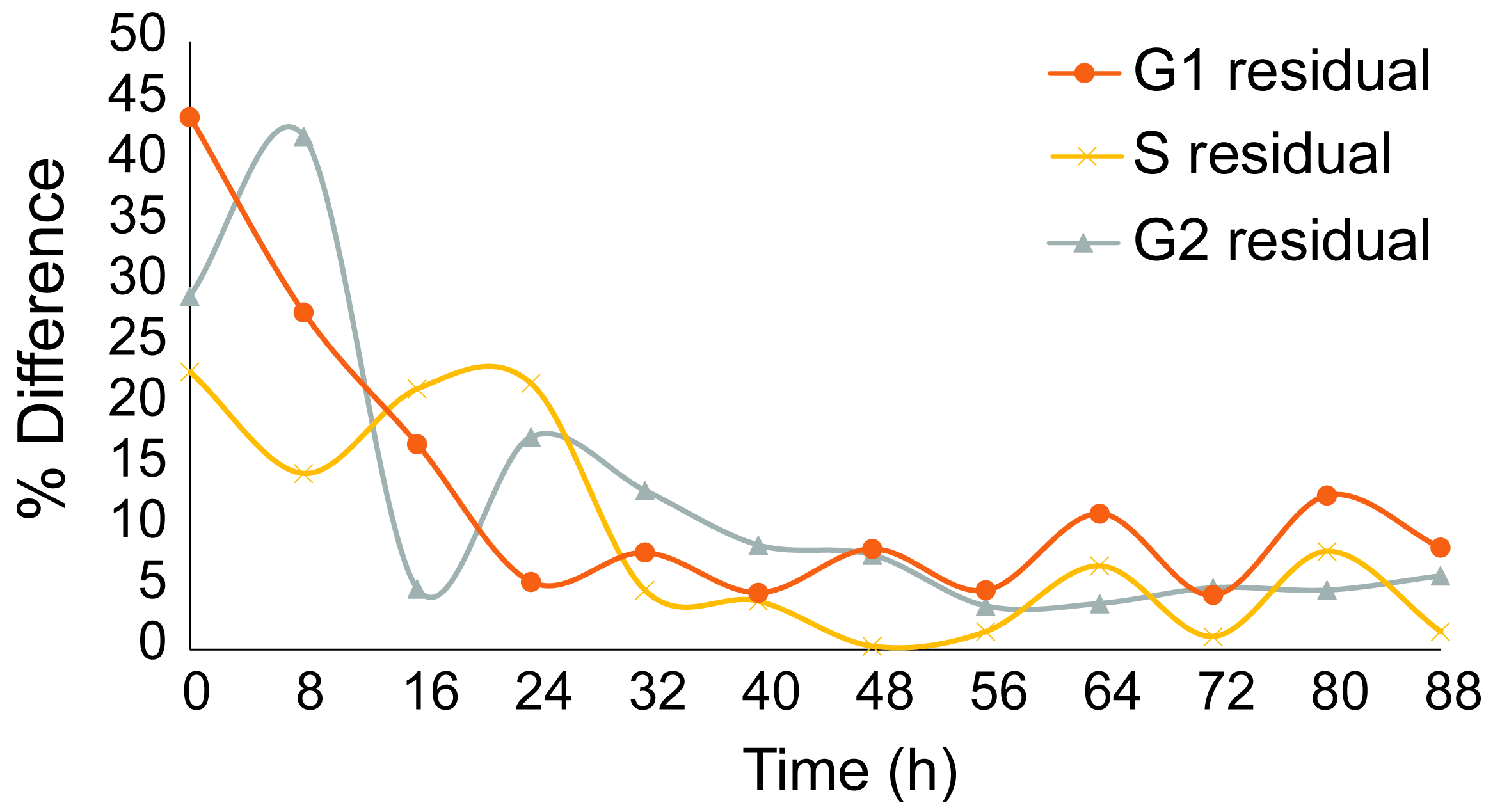

Supplement: S1 Fig — (TIF) [file pcbi.1011080.s001.tif]

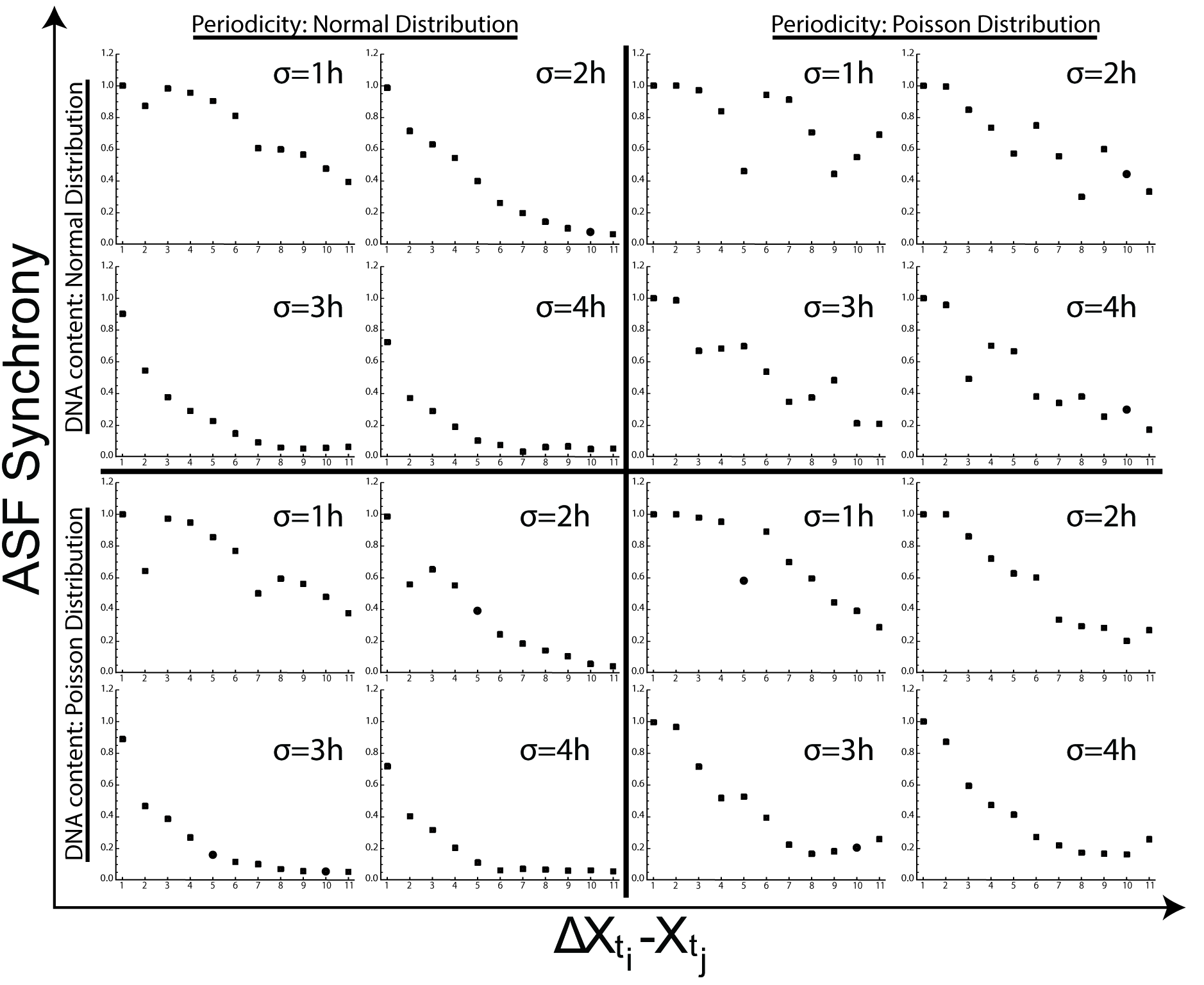

Supplement: S2 Fig — (TIF) [file pcbi.1011080.s002.tif]

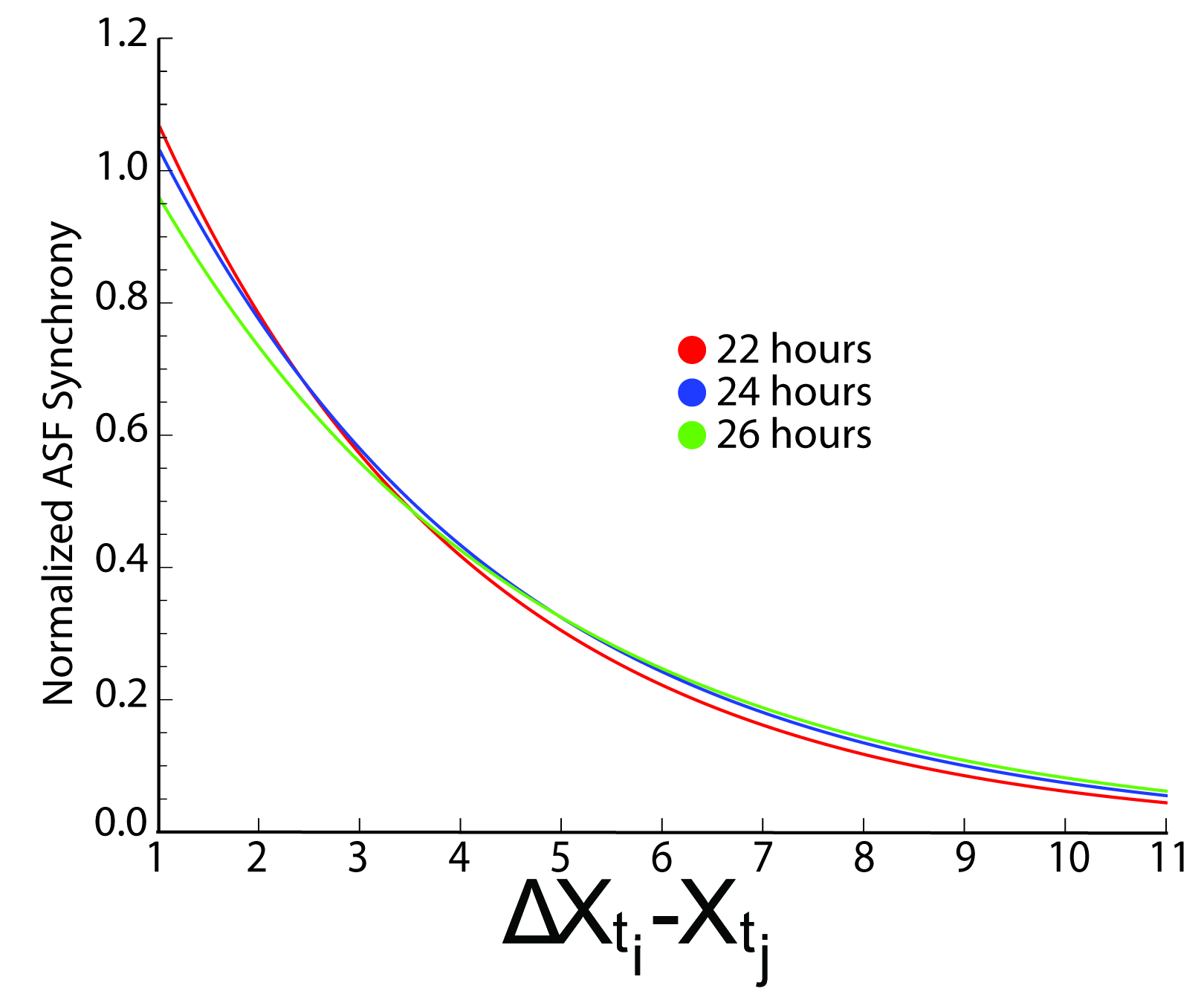

Supplement: S3 Fig — (TIF) [file pcbi.1011080.s003.tif]

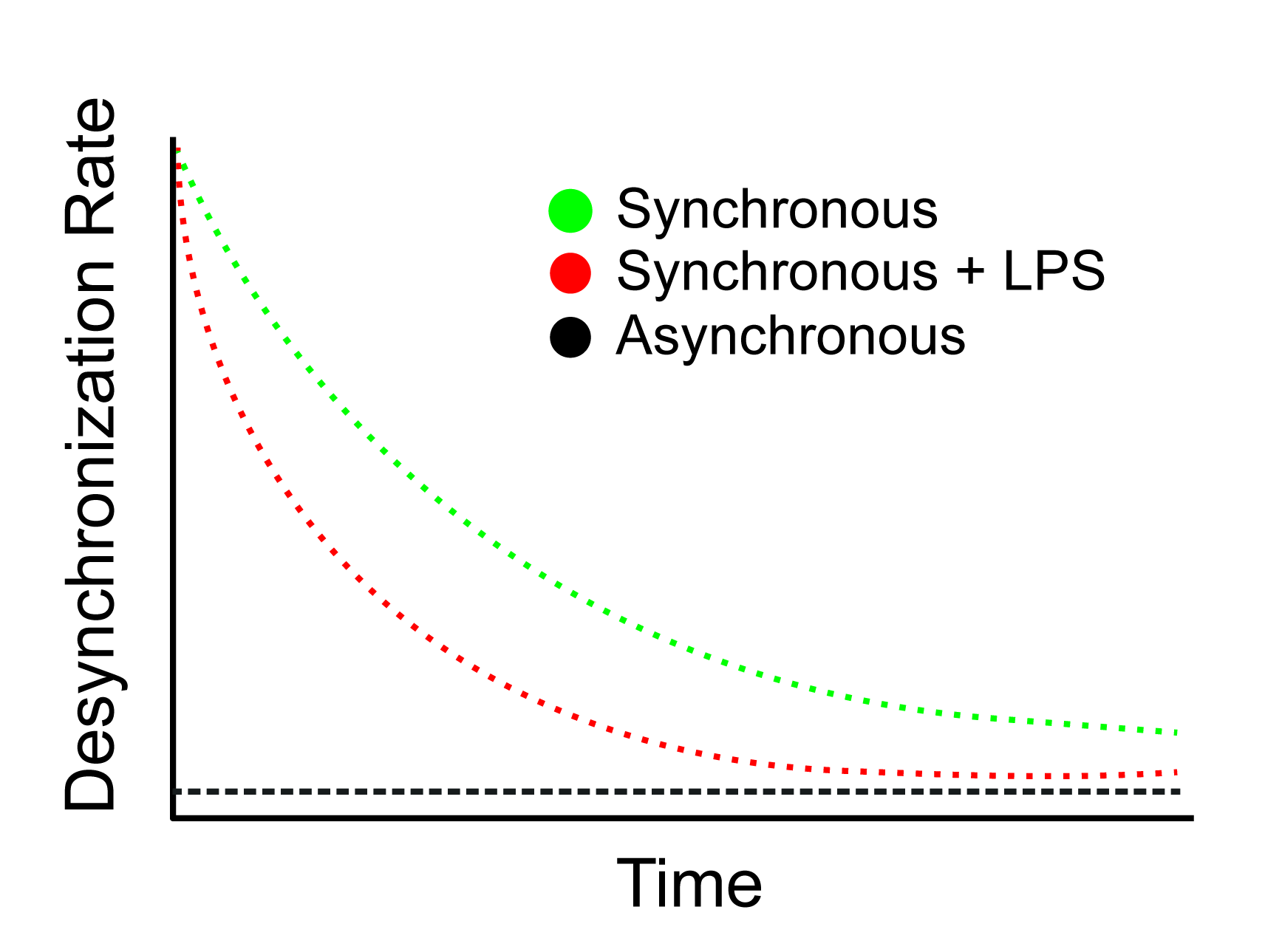

Supplement: S5 Fig — (TIF) [file pcbi.1011080.s005.tif]

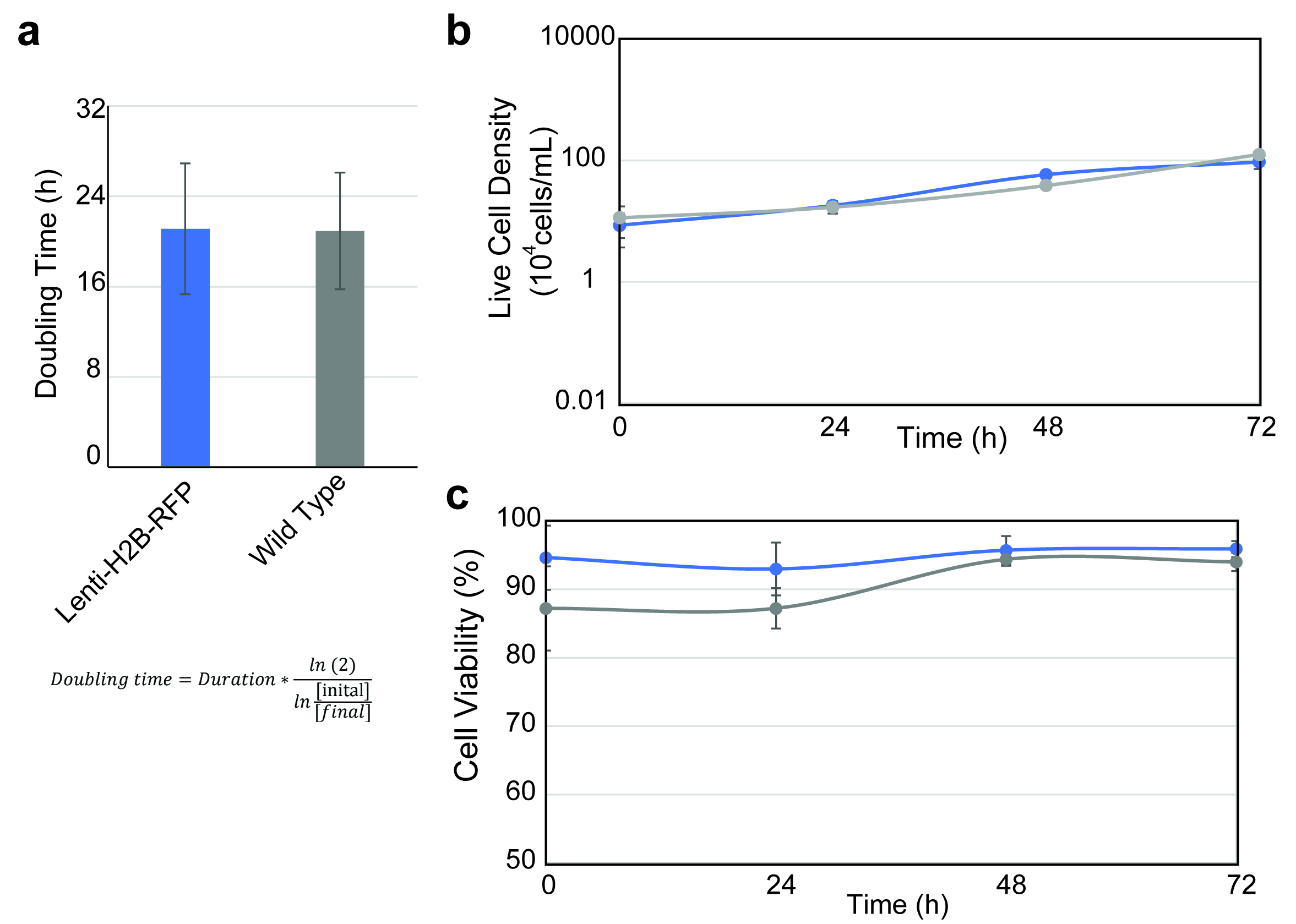

Supplement: S6 Fig — (TIF) [file pcbi.1011080.s006.tif]

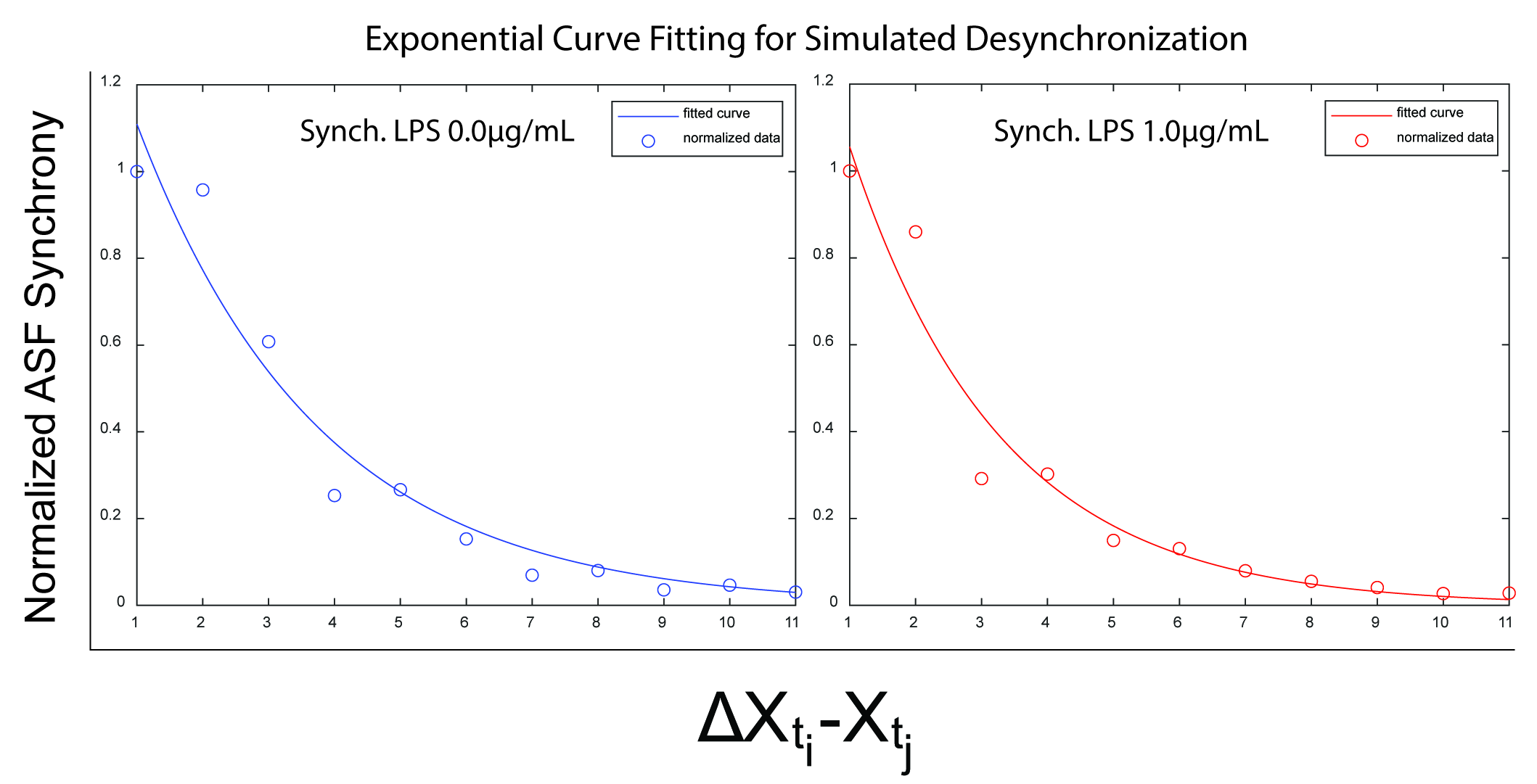

Supplement: S8 Fig — (TIF) [file pcbi.1011080.s008.tif]

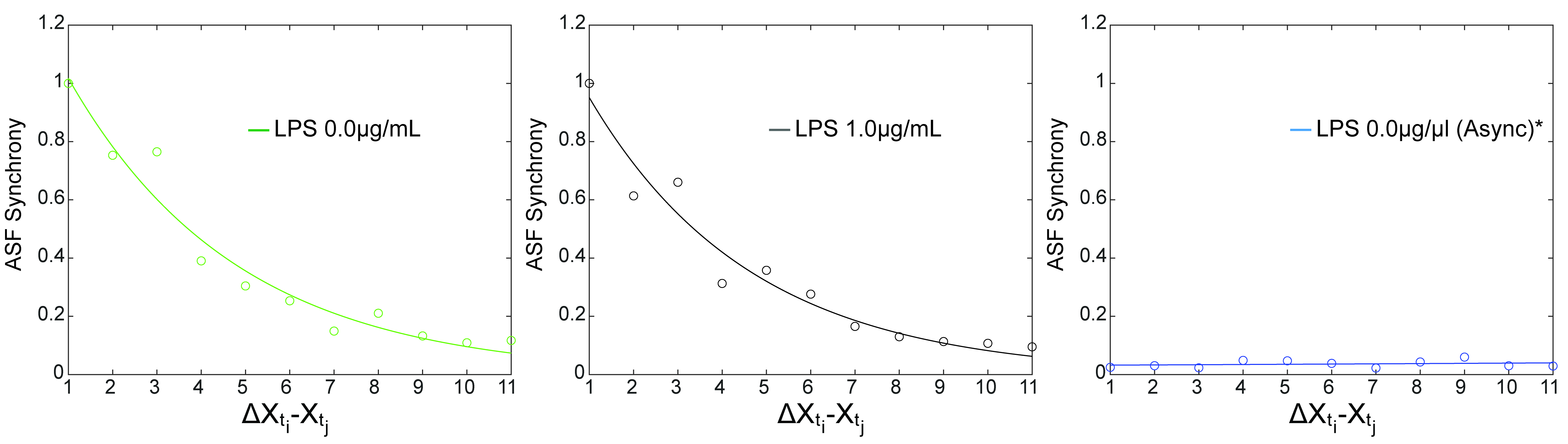

Supplement: S9 Fig — (TIF) [file pcbi.1011080.s009.tif]
