# Supplementary material for: Impact of variability in cell cycle periodicity on cell population dynamics
Source: PLoS Comput Biol. 2023 Jun 20;19(6):e1011080. doi: 10.1371/journal.pcbi.1011080 (PMC10313040; doi:10.1371/journal.pcbi.1011080)
Supplement: Attachment — Submitted filename: Nowak_CC_PLOS_p2p.pdf [file pcbi.1011080.s015.pdf]

Comments to the Authors:
Please note here if the review is uploaded as an attachment.

Reviewer #1:
The paper by Nowak et al. analyzes the effects of variability in the interdivision time on synchrony loss after a cell-cycle arrest, and uses modeling to propose a causal link. The data are of high quality and the conceptual approach is mostly correct. In particular, the application of the Kuiper´s test is interesting and provides a robust statistic to the analysis of cell-cycle synchrony.

The authors initially assumed the duration of S phase to be approximately 1/3 of the interdivision time. However, what the equation reflects is the half-time for DNA replication. As it is, the equation simulates a very short S phase, taking less than 30 min. I would suggest to increase S-phase length to 2-3 h as found by others. This could be attained by decreasing the e base to 1.1 (just a 10% of the interdivision time or so). It would be interesting to see what happens to synchrony loss rates when S-phase duration is increased to experimental levels and a realistic noise is added to the abovementioned base.

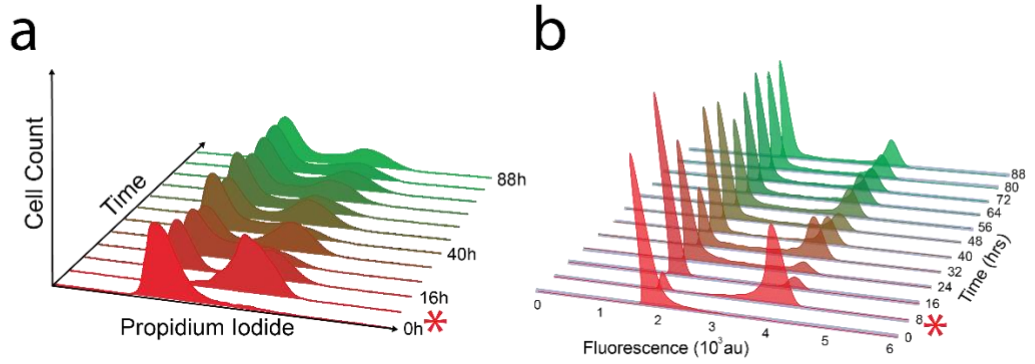

Thank you for the positive and constructive feedback. We apologize for the confusion. The variable s is only used to represent the time at which the cell is halfway through synthesis, not the S-phase itself, which is governed by β and s variables. For a 24-hour period, the S-phase would be roughly 8 hours. As shown in panel **a** in the figure above, experimental observations show that cells take roughly 8 hours to transition between G1 and G2 phases as shown by the two prominent peaks at the 0- and 8-hour timepoints (asterisk). Importantly, running our model shows that our simulated S-phase also takes roughly 8 hours, which can be visualized in panel **b** at the 0- and 8-hour time point (asterisk). We have clarified this in the manuscript.

The paper finishes by showing the effects on synchrony loss when a paradigmatic signaling pathway is affected but, in my view, the direct impact of these data in the main conclusion of the paper is rather limited.

Thank you for your comment. We have included a discussion that highlights the native biological context in which LPS affects cervical cancer cells. Additionally, we have added to the discussion the importance of understanding noise in a cellular population with an emphasis on bioengineering. Also, we believe that our methodology for quantitating cell synchrony as well as its sensitivity to biological noise is an asset that others investigating cellular noise can adopt and expand upon.

Alternatively, fast loss of synchrony after cell division would allow daughter cells to face punctual stressors at different moments of the cell cycle and, hence, increase survival probability. I would suggest the authors to extend the discussion section considering these and other possible scenarios in which their findings would have a relevant functional role.

Thank you for pointing out this intriguing possibility. We have added material discussing the impact synchrony has on individual cells and how cellular noise might be advantageously used in such scenarios.

In summary, although it might have seemed obvious to the non-specialist researcher, the starting hypothesis had not been formally tested and this work will be interesting to those doing research in the interphase between cell proliferation and tissue organization and physiology.

Thank you for your thoughtful responses. We agree. Besides how such a tightly regulated process can rely on stochastic variations, we are now interested in how diseased states such as cancer cells, utilize noise and if noise itself can become a contributor to disease progression. Signaling factors in a tumor microenvironment that confer a higher degree of intercell variability contribute indirectly to tumor cell heterogeneity and associated pathology. An intriguing hypothesis is that cancer cells obtain benefit by having higher noise in cell cycle periodicity, which yields ultra-slow and fast diving cells. This hypothesis opens the path for potential means to exploit variability in cell cycle period for therapeutic purposes.

Reviewer #2: General comments:
In this paper, the authors describe a combination of experiments and simulations to investigate the desynchronization properties in cervical cancer HeLa cells, starting from the G_1/S boundary following double-thymidine block. The authors' main conclusion is that cell cycle desynchronization rate is primarily sensitive to the variability of cell cycle duration. While the authors cover an interesting topic, there are some major concerns that need to be addressed.

Thank you for your time and constructive feedback.

The link between cell cycle duration and (de)synchronization rate seems a straightforward connection, since having the same cell cycle duration is what defines the synchronous behavior within an homogeneous cells population following a block. The authors experimentally show that an increase of cell cycle noise, increases cell cycle variability and desynchronizes the population. The authors indicate that the factors or mechanisms that control cellular desynchronization remains largely unknown and their own phenomenological model is too simple to offer opportunities to explore any of the controlling factors.

We believe that simple phenomenological models are often excellent tools to study biology. We can always increase the complexity of the mathematical model as we gradually incorporate more variables/inputs. Cellular noise occurs at intracellularly and at the cell-to-cell scale, and as such there are plenty of sources responsible for these fluctuations as there are ways in which noise can affect cellular phenotypes. Furthermore, we believe that our investigation into LPS as a small molecule that can impact cellular noise is a novel finding that opens new possible avenues of investigation for studying noise in cervical cancer. Importantly, there exists a native biological context in which LPS can impact cells[1–4] Accordingly, our work provides the initial steps towards understanding factors of noise in cervical cancer cells.

While the model is able to capture the experimental percentages of cells in various phases of the cell cycle, it does not seem to help in advancing the field in understanding the factors that control cellular desynchronization.

Our goal here was to demonstrate experimentally that cellular variability (noise) impacts the rate of desynchronization and to develop a phenomenological model that captures these dynamics. There are no other papers in literature that have performed the same (in any cell type). Accordingly, we respectfully disagree that our work does not advance the field. Noise can be measured and given its role in cell cycle desynchronization, our observation opens the possibility of utilizing/manipulating cellular noise to better observe/control cellular behavior.

In the abstract the authors write that their results highlight an underexplored aspect in cell cycle research (i.e. using desynchronization rate of artificially synchronized in-phase cell populations as a proxy of the degree of variance in cell cycle periodicity), and they themselves leave that idea underexplored by not mentioning anything about this topic in any other part of the paper.

Our last sentence in the abstract is: “Our results show that the desynchronization rate of artificially synchronized in-phase cell populations can be used a proxy of the degree of variance in cell cycle periodicity, an underexplored axis in cell cycle research.” This summarizes the results of our work, and it is a forward-looking statement. We believe our results will inspire work in this direction. To further elaborate on this statement, we added material in the discussion.

The Discussion section is extremely short, and mostly focused on background information about why cell cycle is a crucial process to study, while it fails to explain why the specific ideas from this paper make a significant contribution to the field.

We thank the reviewer for their comment. We have added material in the Discussion section to include the overarching implications in our work of cellular noise in the context of cell cycle periodicity.

The paper may contain experimental processes and methodologies suitable for publications in journals focused on the experimental protocols/methodologies, so if the authors believe that their experimental protocols are novel, we recommend to add more information about that aspect before resubmitting the paper to a different journal.

Quoting the PLOS Computational Biology scope: research articles should demonstrate both methodological and scientific novelty, and provide profound new biological insights, and inclusion of experimental validation of a modest biological discovery through computation does not render a manuscript suitable for PLOS Computational Biology.

While the paper is well written and provides good general background and high-level context on cell cycle research, to publish this manuscript as novel research in PLOS Computational Biology, authors should add information about how the presented model or results advance cell cycle research in a profound way, what is the scientific novelty in this manuscript how their contribution helps advancing the scientific knowledge about the factors that lead to cell cycle variability.

Thank you for your feedback. We hope that our updated manuscript and point-to-point response to the all the comments can persuade this reviewer about the scientific novelty of our work and the general impact to the field.

Minor additional outstanding issues:
- duplicate citation (16, 30)
- please make sure NFkappaB/NFkB nomenclature is consistent

We thank the reviewer for pointing these typos out, and they have corrected in the manuscript.

Reviewer #3:
The goal of this manuscript is to investigate the desynchronization of human cells arrested in the same phase of their cell cycle. The authors propose the autosimilarity function (ASF), an elegant measure of cell cycle asynchrony, based on the cumulative distribution of cellular DNA amount. The measure is equivalent to the Kuiper two-sample test statistic. Next, a phenomenological mathematical model of DNA accumulation in cycling cells augmented with a stochastic term can produce ASF time-dependence as in the experiments. The model predicts that increasing the noise should accelerate desynchronization, which is verified experimentally by using lipopolysaccharide (LPS) to increase the noise of cell cycle periods.

Overall, this is an elegant, clearly presented, relatively simple yet interesting study that deserves publication. The manuscript should benefit from the authors addressing the following comments.

Thank you for your time, feedback, and constructive comments.

(1) Figure 1D: axis label and units are missing. An axis label would also be needed for panels 1b and 1c. Please ensure that all plots have axis labels and units.

Thank you for pointing out this error. We have made the corrections in the manuscript.

(2) At least initially, the ASF might depend on the measurement intervals. For example, if accidentally the ASF was measured exactly at the time points where the red and green lines cross each other in Figure 1d, the ASF would be lower. It would be useful to include a rationale for choosing an optimal time interval for ASF measurements. This optimal interval probably depends on the cell cycle period, right?

We thank the reviewer for this constructive comment. Our rationale for choosing 8-hour intervals was based on the length of cell cycle phases and planning for frequent measurements for the duration of the experiment (88 hours). Given how quickly cells can desynchronize following release from cell cycle arrest, we reasoned that optimal starting point for our ASF analysis was one that showed the greatest change in DNA content (2n and 4n) and one in which the populations being compared maintained sufficient synchrony between timepoints. Our initial PI measurements of double thymidine blocked cells revealed that S-phase was completed after 8 hours, agreeing with previous reported values [ref]. We therefore chose the 8-hour interval for subsequent experiments as it allowed us to capture the greatest change in DNA distributions while initial synchrony was still maintained.

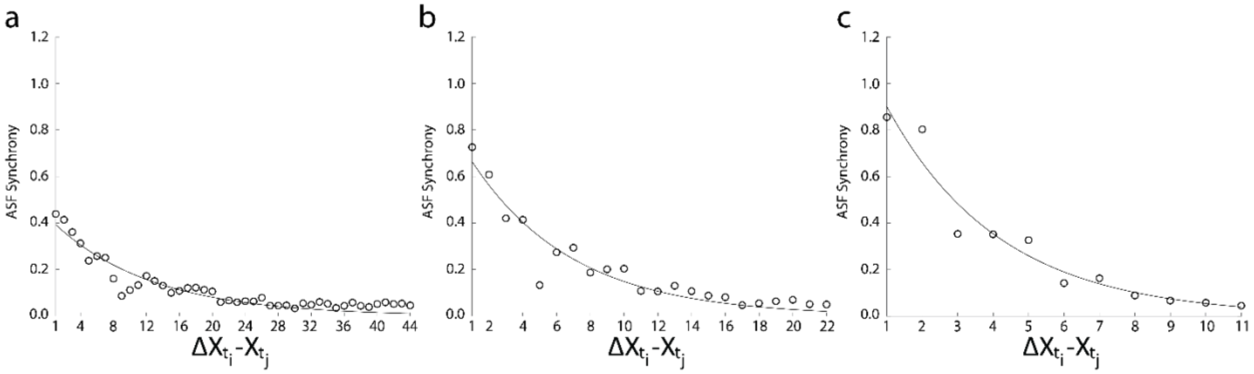

Driven by the reviewer's comment, as shown in the figure above, we ran our simulations for 2-, 4-, and 8-hour intervals for the 88-hour measurement. Shown in the figure above are the raw KS scores for each measurement interval tested. As expected, more frequent measurements result in smaller differences in observable DNA content change between pairwise comparisons, which results in lower changes in KS. Interestingly, the point of desynchronization does not change significantly with each interval reaching asynchronicity after the second doubling event and before the third doubling event (~56-64 hours). This may give an opportunity to measure more frequently to extract information. Thank you for pointing this property.

(3) Do the data points in Figure 2c have error bars? It would be interesting to think of a statistical test for cells reaching asynchrony. This would require repeated measurements of ASF and testing if the ASF values of an initially synchronous population are significantly different from an asynchronous population's ASF values.

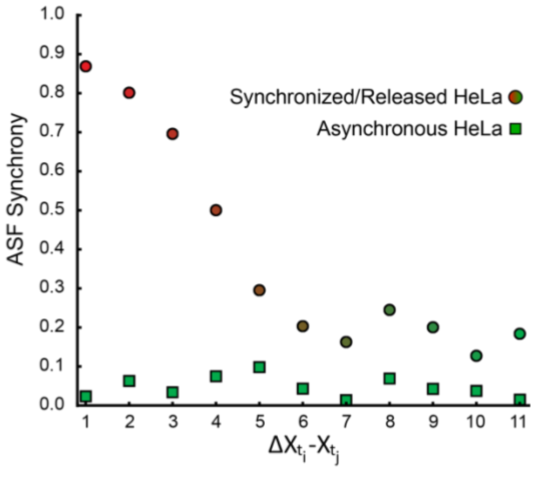

We thank the reviewer for their comment. There are no error bars in Figure 2c as this is a single experiment. We have performed 3 independent runs of HeLa cells blocked at G_1/S boundary and measured at 8h intervals for 88 hours. These were not technical replicates, but experiments performed at different times and are averaged in Figure 4e. Indeed, we cannot definitely calculate when a synchronous population reaches full asynchrony using PI stain measurements at 8-hour intervals. We suspect that full asynchrony can we approximated to be between intervals of ASF synchrony values that stop moving monotonically. As the reviewer states, to properly investigate this (and provide a statistical test) we would have to perform repeated measurements of ASF at different intervals.

(4) In Supplementary Figure 2, the effect of values selected from a normal versus Poisson distribution are compared. However, many Poisson distributions exist, depending on the distribution's parameters. The parameters should be specified for both normal and Poisson distributions. Trying multiple parameters would be useful. For some parameter choices, the Poisson results should tend to be similar to the results obtained using normal-distributed values.

We thank the reviewer for pointing out this confusion. Our simulations were run with a starting value of 24 hours before our error term is applied, which then generates a new period for each cell based on the applied distribution. We simulated 4 cases for lambda (1-4) that yield noise values bounded to 0-4 hours, which are physiologically relevant, and produce typical Poisson distributions. The higher lambda cases do resemble normal distributions, but, accordingly, were not included as they generated nonphysiologically relevant values.

(5) Equation 5 and Figure 3: what aspects of the DNA synthesis does the noise term affect? Is it only the time of the uprise from dna0? Or also the slope of the rise?

We thank the reviewer for their comment. It is applied to the initial DNA content as well as the entire period. The slope shouldn't be impacted by the longer or shorter variance times for period as it applied to the whole cell cycle. No one phase is being directly impacted, all are being proportionately impacted. We have clarified this in the manuscript.

(6) Is there a stronger justification for using the model in Equations 2 – 5 besides the shape of DNA accumulation over time? Are there other models of DNA accumulation versus cell cycle time in the literature? It would be useful to discuss this to understand the novelty of the approach in the context of other papers.

We thank the reviewer for their comment. Our justification for using these equations was to develop a simple phenomenological model, that includes period and DNA content as variables and recapitulates the experimental results, which essentially map cell cycle stage to propidium iodide staining of DNA.

The literature on similar mathematical models that investigate DNA accumulation versus time do not rely on solely on extracting DNA content from PI staining. Most instances will couple PI with an additional labeling agent or assume DNA content based on cell cycle stage and investigate transcriptional levels of known cell cycle regulators to build models. We have included additional text that discusses other instances of mathematical modeling in cell cycle analysis, some of which include DNA accumulation over time.

(7) "RelA, have shown to interact…", probably a "have been" was intended here.

Thank you for pointing this out. We have made the corresponding changes in the manuscript.

(8) While the LPS treatment increases the noise of cell cycle times, it seems to also affect their mean. Ideally, the average cell cycle time should stay unchanged. If not, then the change in the mean cell cycle time may affect the rate of desynchronization. This should be tested by modeling, doing a parameter scan for tau.

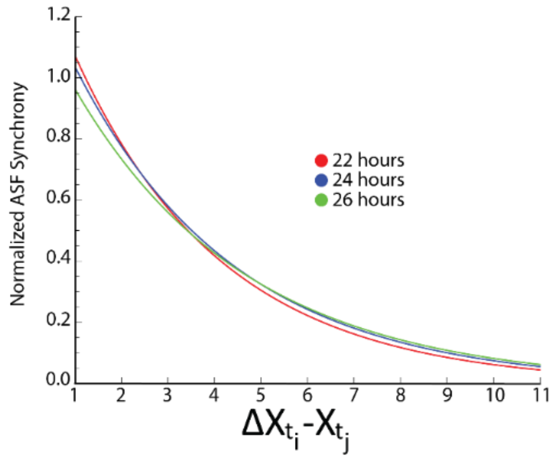

We thank the reviewer for their comment. Indeed, ideally LPS administration would only impact the variation. However, testing our model shows differing means did not reveal a significant impact on desynchronization rate when the populations tested had the same variance (3 hours). Please see the above figure, which we have added to the supplemental material (**Supplementary Figure 3**).

(9) It would be useful to develop a metric of how "fast" a cell population reaches asynchrony, and then apply it to Figure panels 4c, 4e. Yes, one curve is always below the other, but they start the same way, one below the other. So, it is like declaring a runner as the winner after giving him a head start. Would exponentials fit these curves? Could the exponent be a metric for the speed of approaching asynchrony?

We thank the reviewer for their comment. The data points in Figure 4e are averaged then normalized to their starting value, which means all samples initially start from 1. We then fit the data points to an exponential trend, and indeed, lower exponents values coincide with increased rates of desynchronization. So potentially, the exponent can be used to show the change in the rate of desynchronization across conditions, but as we do not have a clear cutoff value for asynchronous populations we chose to simply show plots to convey the approach to asynchrony. However, this will be further investigated in future experiments.

(10) Regarding the independent effects of the noise and the mean, experimental approaches have been developed for their decoupled control (meaning that the noise changes while the mean does not). Prior work on this may be worth mentioning, see PMID:17189188 and PMID:31235692.

We thank the reviewer and greatly appreciate the feedback towards improving our work. We have accordingly added these citations to our manuscript.

References

1. Jiang, X., Yuan, J., Dou, Y., Zeng, D. & Xiao, S. Lipopolysaccharide Affects the Proliferation and Glucose Metabolism of Cervical Cancer Cells Through the FRA1/MDM2/p53 Pathway. *Int. J. Med. Sci.* **18**, 1030 (2021).
2. Hao, H. *et al.* Lipoxin A4 Suppresses Lipopolysaccharide-Induced Hela Cell Proliferation and Migration via NF-κB Pathway. *Inflammation* **38**, 400–408 (2015).
3. Wang, J. *et al.* Cancer-derived immunoglobulin G promotes LPS-induced proinflammatory cytokine production via binding to TLR4 in cervical cancer cells. *Oncotarget* **5**, 9727–9743 (2014).
4. You, L. *et al.* Inhibition of HMGB1/RAGE axis suppressed the lipopolysaccharide (LPS)-induced vicious transformation of cervical epithelial cells. *Bioengineered* **12**, 4995 (2021).

Have the authors made all data and (if applicable) computational code underlying the findings in their manuscript fully available?
The PLOS Data policy requires authors to make all data and code underlying the findings described in their manuscript fully available without restriction, with rare exception (please refer to the Data Availability Statement in the manuscript PDF file). The data and code should be provided as part of the manuscript or its supporting information, or deposited to a public repository. For example, in addition to summary statistics, the data points behind means, medians and variance measures should be available. If there are restrictions on publicly sharing data or code —e.g. participant privacy or use of data from a third party—those must be specified.
Reviewer #1: Yes
Reviewer #2: Yes
Reviewer #3: Yes

PLOS authors have the option to publish the peer review history of their article (what does this mean?). If published, this will include your full peer review and any attached files.

If you choose “no”, your identity will remain anonymous but your review may still be made public.

Do you want your identity to be public for this peer review? For information about this choice, including consent withdrawal, please see our Privacy Policy.
Reviewer #1: No
Reviewer #2: No
Reviewer #3: No

Figure Files:
While revising your submission, please upload your figure files to the Preflight Analysis and Conversion Engine (PACE) digital diagnostic tool, https://pacev2.apexcovantage.com. PACE helps ensure that figures meet PLOS requirements. To use PACE, you must first register as a user. Then, login and navigate to the UPLOAD tab, where you will find detailed instructions on how to use the tool. If you encounter any issues or have any questions when using PACE, please email us at figures@plos.org.
Data Requirements:
Please note that, as a condition of publication, PLOS' data policy requires that you make available all data used to draw the conclusions outlined in your manuscript. Data must be deposited in an appropriate repository, included within the body of the manuscript, or uploaded as supporting information. This includes all numerical values that were used to generate graphs, histograms etc.. For an example in PLOS Biology see here:
http://www.plosbiology.org/article/info%3Adoi%2F10.1371%2Fjournal.pbio.1001908#s5.
Reproducibility:
To enhance the reproducibility of your results, we recommend that you deposit your laboratory protocols in protocols.io, where a protocol can be assigned its own identifier (DOI) such that it can be cited independently in the future. Additionally, PLOS ONE offers an option to publish peer-reviewed clinical study protocols. Read more information on sharing protocols at
https://plos.org/protocols?utm_medium=editorial-email&utm_source=authorletters&utm_campaign=protocols
